# Overexpression of Mcl-1L Splice Variant Is Associated with Poor Prognosis and Chemoresistance in Oral Cancers

**DOI:** 10.1371/journal.pone.0111927

**Published:** 2014-11-19

**Authors:** Vinayak Palve, Sanchita Mallick, Gauri Ghaisas, Sadhana Kannan, Tanuja Teni

**Affiliations:** 1 Teni Lab, Advanced Centre for Treatment, Research and Education in Cancer (ACTREC), Tata Memorial Centre, Kharghar, Navi Mumbai-410210, India; 2 Epidemiology and Clinical Trial Unit (ECTU), Advanced Centre for Treatment, Research and Education in Cancer (ACTREC), Tata Memorial Centre, Kharghar, Navi Mumbai-410210, India; Indian Institute of Science, Bangalore, India

## Abstract

**Background:**

Altered expression of Mcl-1, an anti-apoptotic member of the Bcl-2 family, has been linked to the progression and outcome of a variety of malignancies. We have previously reported the overexpression of Mcl-1 protein in human oral cancers. The present study aimed to evaluate the clinicopathological significance of the expression of three known Mcl-1 isoforms in oral tumors and the effect of targeting Mcl-1L isoform on chemosensitivity of oral cancer cells.

**Methods:**

The expression of Mcl-1 isoforms- Mcl-1L, Mcl-1S & Mcl-1ES was analyzed in 130 paired oral tumors and 9 oral cell lines using quantitative real-time PCR & protein by western blotting. The Mcl-1 mRNA levels were correlated with clinicopathological parameters and outcome of oral cancer patients. The effect of Mcl-1L shRNA or Obatoclax (a small molecule Mcl-1 inhibitor), in combination with Cisplatin on chemosensitivity of oral cancer cells was also assessed.

**Results:**

Anti-apoptotic Mcl-1L was predominantly expressed, over low or undetectable pro-apoptotic Mcl-1S and Mcl-1ES isoforms. The Mcl-1L transcripts were significantly overexpressed in all cancer cell lines and in 64% oral tumors versus adjacent normals (*P*<0.02). In oral cancer patients, high Mcl-1L expression was significantly associated with node positivity (*P* = 0.021), advanced tumor size (*P* = 0.013) and poor overall survival (*P* = 0.002). Multivariate analysis indicated Mcl-1L to be an independent prognostic factor for oral cancers (*P* = 0.037). Mcl-1L shRNA knockdown or its inhibition by Obatoclax in combination with Cisplatin synergistically reduced viability and growth of oral cancer cells than either treatment alone.

**Conclusion:**

Our studies suggest that overexpression of Mcl-1L is associated with poor prognosis and chemoresistance in oral cancers. Mcl-1L is an independent prognostic factor and a potential therapeutic target in oral cancers.

## Introduction

Oral cancer is the most prevalent cancer among Indian males and is predominantly associated with tobacco-chewing habit prevalent in the country [1]. Despite recent advances in treatment modalities like surgery, radiotherapy/chemotherapy, the long term survival of oral cancer patients has not changed significantly. The factors associated with poor prognosis of oral cancer include, presentation at an advanced clinical stage & uncontrolled loco-regional recurrence [2]. Hence it is important to elucidate the mechanisms involved in the development and progression of oral cancer and identify molecular targets for better disease management.

Oral cancers have repeatedly been associated with apoptotic dysregulation [3]. The pro and anti-apoptotic members of the Bcl-2 family are the key regulators of cellular apoptosis and play a critical role in regulating cell survival [4]. Mcl-1 (Myeloid cell leukemia-1) is an important anti-apoptotic member of the Bcl-2 gene family, essential for development, differentiation, and proliferation [5]. Cellular expression of Mcl-1 is tightly regulated through multiple transcriptional and post-transcriptional mechanisms [6]. Increased Mcl-1 expression can produce moderate short-term viability enhancement in a broad range of cell types. Mcl-1 may promote cell survival by suppressing the release of cytochrome-c from mitochondria via heterodimerisation and the neutralization of effector pro-apoptotic BH3-only proteins such as Bak and Noxa [7]. The overexpression of Mcl-1 has been reported in a variety of malignancies including hematopoietic, lymphoid and solid tumors [8], [9]. Overexpression of Mcl-1 has been associated with aggressive tumor features, resistance to treatment and poor prognosis in breast, gastric, ovarian & cervical cancers [10]–[13].

Although the Mcl-1 gene has been studied extensively in multiple myeloma and leukemia, there are rare reports on Mcl-1 analysis in head and neck cancer. Recent studies from our laboratory have demonstrated significant overexpression of Mcl-1 protein in oral cancer cell lines, premalignant lesions (OSF) and oral tumors by immunohistochemistry [14]. We have also demonstrated high PCNA and Mcl-1 protein expression to be associated with poor prognosis in oral cancer patients treated with definitive radiotherapy [15]. However, the situation is complex due to the existence of three distinct Mcl-1 isoforms having contrasting functions namely anti-apoptotic Mcl-1L and pro-apoptotic Mcl-1S & Mcl-1ES [16]. Interestingly, our lab has recently reported the association of anti-apoptotic Mcl-1L isoform with survival and radioresistance of oral squamous carcinoma cells [17].

Mcl-1 has also been shown to play a role in chemoresistance of a variety of cancers, but the role of its isoforms in chemoresistance has not been studied in cancers including oral cancers. Radiation followed by chemotherapy is the common treatment modality for oral cancer and Mcl-1 overexpression has been shown to provide resistance to conventional chemotherapeutic drugs like Cisplatin [18]. Several reports have shown that Mcl-1 promotes cell survival and targeting Mcl-1 via BH3-mimetic molecules can induce cell death in Cisplatin resistant cancer cells [19], [20]. Recently, Obatoclax, a BH3 mimetic small molecule inhibitor has been shown to antagonize Mcl-1 protein and overcome Mcl-1 mediated resistance to apoptosis [21], [22]. Our recent studies indicate Mcl-1 to be important for the survival of oral cancer cells and hence targeting Mcl-1 could be useful in treatment of oral cancer patients. However, to the best of our knowledge, such studies are not available in oral cancer. The present study was thus undertaken to evaluate the clinical significance of Mcl-1 isoforms in oral cancer and the efficacy of targeting Mcl-1 to chemosensitize oral cancer cells *in vitro*.

## Materials and Methods

### Cell cultures

Seven oral cancer cell lines- SCC25, QLL1 (From Dr. Park, Yonsei University College of Medicine, Korea [23] & Dr Rheinwald, Harvard Medical School, Boston [24]); UPCI:SCC029B, UPCI:SCC040 & UPCI:SCC074 (From Dr. S Gollin, University of Pittsburgh, PA) [25]; AW8507 & AW13516 [26] were used in the study. Additionally an immortalized Fetal Buccal Mucosa (FBM) [27] and a Dysplastic Oral Keratinocyte (DOK) (from Dr. Ken Parkinson, Queen Mary's School of Medicine & Dentistry, UK)[28] cell lines were also used in study. The cells were maintained in MEM or IMDM supplemented with 10% FBS & 1% standard antibiotic mixture in 5% CO_2_ incubator at 37°C as described earlier [17], [23], [29].

### Oral tissues

This study was approved by the Institutional Review Board of Tata Memorial Centre and Sharad Pawar Dental College, India. Written informed consents were obtained from all the study participants. Treatment naïve primary oral tumor samples and their adjacent normal tissues were obtained from 130 patients undergoing surgery at the Head and Neck Unit and from the ICMR National Tumor Tissue Repository, Tata Memorial Centre, Mumbai, India. Inflamed oral tissues (n = 20) were collected as healthy normal tissues from patients undergoing minor dental surgical procedure at the Department of Oral Pathology, Sharad Pawar Dental College, Wardha, India. All the tissues were frozen immediately in liquid nitrogen and stored at −80°C until analysis. The clinicopathological characteristics of the cohort are illustrated in Table 1.

**Table 1:** Clinicopathological characteristics of 130 oral cancer patients.

Characteristics	No. (%)
*Gender*	
Male	99 (76.2)
Female	31 (23.8)
*Age* (Range 13–80 years)	
>53	63 (48.5)
<53	67 (51.5)
*Habits*	
Tobacco chewing	81 (62.3)
Tobacco chewing + Alcohol	15 (11.5)
Smoking	04 (3.1)
Smoking + Alcohol	02 (1.5)
Tobacco chewing + Smoking	13 (10)
Tobacco + Smoking + Alcohol	06 (4.6)
No habits	07 (5.4)
NA	02 (1.6)
*Primary site of tumor*	
Buccal Mucosa	46 (35.4)
Tongue	45 (34.6)
Alveolus	35 (26.9)
Others	04 (3.1)
*Tumor classification*	
T1 + T2	42 (32.3)
T3 + T4	88 (67.7)
*Lymph Node*	
N0	47 (36.2)
N1	32 (24.6)
N2	51 (39.2)
*Differentiation*	
Well	16 (12.4)
Moderate	82 (63.6)
Poor	31 (24.0)

Abbreviations: NA  =  Not Available.

### RNA isolation and Real-time PCR

RNA from cell pellets and tissues were extracted using TRI reagent (Sigma, USA) according to the manufacturer's protocol. The RNA was dissolved in DEPC-treated water and contaminating DNA was removed by DNaseI treatment (Sigma, USA). RNA integrity was analyzed by electrophoresis and samples were preserved at −80°C until analysis, as described earlier [17]. cDNA was synthesized with 500 ng total RNA, using a First Strand cDNA synthesis kit (MBI Fermentas, Canada) according to the manufacturer's instructions. The cDNA was then subjected for quantitative real-time PCR using Taqman universal PCR master mix (ABI, 4304437) and Mcl-1 isoform specific gene expression probes (ABI, *Mcl-1L*:Hs00172036_m1; *Mcl-1S*:Hs00766187_m1 & *Mcl-1ES*:4331348). The amplification was done on ABI-7900HT real time PCR system (Applied Biosystems, USA). GAPDH (ABI, Hs99999905_m1) was used as internal control to normalize inter sample variation in RNA input & amplification efficiency. All amplification reactions were done in triplicates, using DEPC treated water as negative controls. The analysis of gene expression data was done by using the Comparative C_T_ method of relative quantification [30].

### Western blotting

Proteins were extracted from cell pellets and tumor tissues as described earlier [14]. Briefly, 30 mg of frozen tissue was pulverized in mortal & pestle using liquid Nitrogen, dissolved in chilled ProteoJET Mammalian Cell Lysis Reagent containing ProteoBlock Protease Inhibitor Cocktail (Fermentas, USA) and sonicated on ice. The tissue or cell lysates were cleared by centrifugation at 14000 rpm at 4°C. The protein estimation was performed by the Bradford method. The tissue/cell lysates (30–50 µg) were resolved on 12% SDS-PAGE gels and transferred onto PVDF membranes (Millipore, USA). Membranes were blocked with 5% skimmed milk in TBS for 2 hrs. and incubated overnight at 4°C with mouse monoclonal antibody against Mcl-1L (1∶1000) (Santa Cruz Biotechnology, USA). The membranes were stripped and reprobed with rabbit polyclonal antibody against ß-actin (1∶2000) (Santa Cruz Biotechnology, USA) as loading control. Secondary antibodies used were Horseradish peroxidase conjugated IgG (1∶5000) (Santa Cruz Biotechnology, USA). Proteins were visualized with enhanced chemiluminescence kit (GE Healthcare, US). Densitometry analysis of developed X-ray film was performed using ImageJ software (NIH, Bethesda, MD).

### Mcl-1L knockdown

The expression of Mcl-1L was downregulated by cloning Mcl-1L shRNA cassette in pTRIPZ lentiviral system as per manufacturer's instructions (Applied Biosystems, USA). A non-targeting oligonucleotide sequence was cloned as control. The viral particles were generated by co-transfecting the recombinant Mcl-1LshRNA-pTRIPZ constructs and packaging plasmids in HEK293FT cells. Further, three oral cancer cell lines namely AW8507, UPCI:SCC029B & UPCI:SCC040 were transduced and the stable selection was done using puromycin selection marker. The expression for shRNA was induced by doxycycline and post 72 hrs. of treatment, the levels of Mcl-1L were assessed by qRT-PCR and western blotting. The specific silencing of Mcl-1L was confirmed in three independent experiments.

### Cell death by PI staining

PI (Propidium Iodide, Santa Cruz Biotechnology, CA) staining was done for the detection of cell death in the three oral cancer cell lines (AW8507, UPCI:SCC029B & UPCI:SCC040) after different treatment combinations. Briefly, cells were collected by trypsinization, post different treatments as described earlier. Cells were washed with PBS and 10 µl PI was added. The cells were then incubated for 2 min in dark and analyzed at 488 nm, on a flow cytometer (FACS Caliber, BD, USA).

### MTT assay

Cells were seeded at 2000 cells per well in 96-well plates containing DMEM with 10% FBS. After 24 h, cells were either left untreated or treated with different concentrations of Cisplatin (0.025–10.0 µg/ml) (MP Biomedicals, India) or Obatoclax (0, 1.0–10.0 nM) (Selleck chem USA). The 50% inhibitory concentrations (IC50) for Cisplatin and Obatoclax were calculated from the survival curves for all the cell lines. DMSO containing medium served as the respective control. Following incubation of cells with Obatoclax and/or Cisplatin for 72 h, the cell growth was assessed by the MTT assay, as per manufacturer's instructions (Sigma, USA). Briefly, the spent medium was removed and 50 µl of MTT solution (1 mg/ml) was added to each well. The plates was incubated for 4 hrs at 37°C in CO_2_ incubator and the formazan crystals in viable cells were dissolved by using dimethyl sulfoxide (DMSO, 100ul per well). The plate was agitated on an orbital shaker for 10 min and the absorbance was measured at 540 nm using reference wavelength of 690 nm on micro plate reader (Spectramax, USA). Five wells were used for each drug concentration, and the experiment was repeated three times. In another experiment, to evaluate whether combination of treatments would achieve higher growth inhibition than individual treatment, oral cancer cells were treated with doxycycline to knockdown Mcl-1 expression or Obatoclax (5 nM) to inhibit Mcl-1 activity, followed by Cisplatin at a concentration lower than the IC50 dose. All the experiments were performed in triplicates.

### Trypan blue assay & Confocal Microscopy

Cells were seeded into 24-well plates at a density of 5×10^4^ per well. The Mcl-1 expression was targeted either by Doxycycline or Obatoclax treatments or in combination with Cisplatin as described above. Cells were trypsinzed after 48 hrs. of treatment and trypan blue (0.4%) staining was performed. The number of viable cells were counted using a hemocytometer and compared to untreated control. The data are shown from three independent experiments. The imaging of cells after different treatments was carried out using an inverted microscope with LSM Image Browser 4.2 software (Carl Zeiss, USA).

### Statistical analysis

Statistical analysis was done using a licensed version of SPSS software package 15.0. To statistically correlate the expression of Mcl-1 isoforms with clinicopathological parameters, the data was dichotomized into two groups namely: the Mcl-1 isoform high expressers and low expressers. For comparison the mean expression of Mcl-1 isoforms in healthy normals and histologically normal adjacent tissues were used. The correlation between clinicopathological parameters (gender, age, site, habits, size, node, metastasis, recurrence) was done using the Chi Square test (χ^2^). Kaplan Meier curves were used for evaluating the overall survival and the difference within the groups was calculated with Log Rank test of significance. The predictive parameters in the univariate analysis were incorporated into multivariate analysis using Cox's proportional hazard test to identify the factors that were independent predictors of survival. The Graphpad Prism5 software was used to plot graphs and the data of two similar & dissimilar groups were statistically analyzed by Student-t test & Mann Whitney test. The variation among group means was analyzed by one-way analysis of variance (ANOVA) test. The data are reported as mean ±SD. The p value <0.05 was considered statistically significant.

## Results

### Expression of Mcl-1 isoforms in oral cell lines

**Figure 1:**
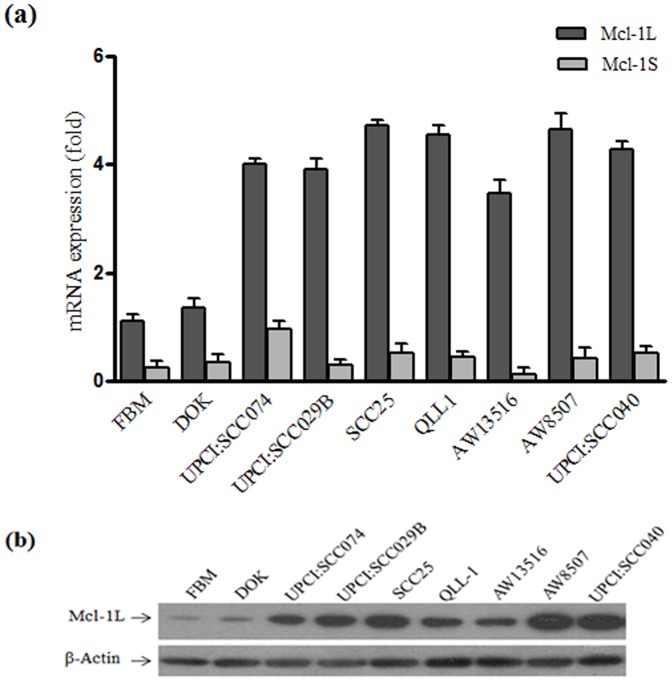
Expression of Mcl-1 isoforms in oral cell lines. (a) Expression of Mcl-1L & Mcl-1S mRNA in oral cell lines; Mcl-1 ES levels were undetectable (* P≤0.03) (b) Mcl-1L protein expression in oral cell lines.

qRT-PCR analysis revealed, predominant high expression of anti-apoptotic Mcl-1L isoform over low or undetectable pro-apoptotic Mcl-1S and Mcl-1ES expression in all the oral cell lines (Figure1a). Mcl-1L was found to be significantly overexpressed at both mRNA & protein level in all seven oral cancer cell lines (SCC25, UPCI:SCC029B, UPCI:SCC040, UPCI:SCC074, QLL1, AW8507 and AW13516) as compared to immortalized normal FBM & dysplastic DOK cell line (Figure 1).

### Expression of Mcl-1 isoforms in oral tissues

**Figure 2:**
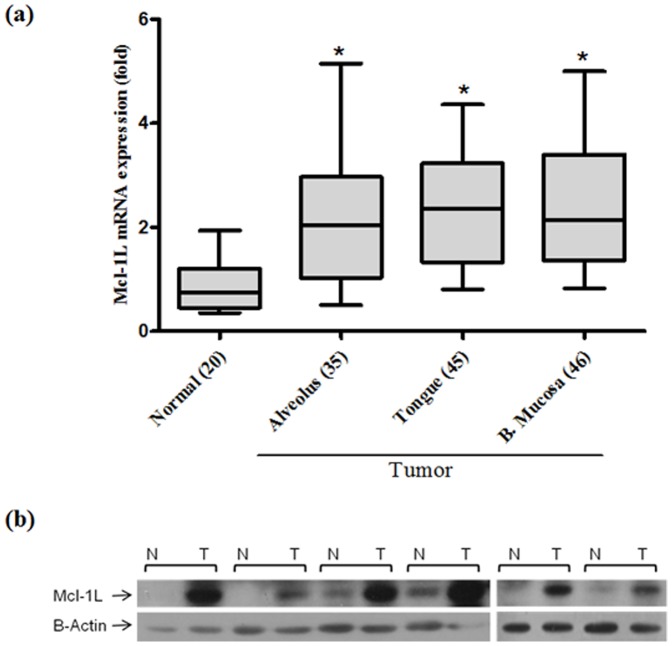
Correlation of Mcl-1L mRNA expression in oral normal versus tumor tissues. (a) Expression of Mcl-1L mRNA in normal vs. oral tumors of different subsites (* P≤0.02); (b) Mcl-1L protein expression in adjacent normal versus tumors.

qRT-PCR analysis revealed significant (p≤0.02) high expression of Mcl-1L mRNA in 64% (84/130) of oral tumors of different subsites as compared to normal tissue (Figure 2a). Moreover, no significant difference was observed between Mcl-1L expression from tumors of different subsites. The relative expression of Mcl-1L isoform was ∼5 fold higher than Mcl-1S and ∼10 fold higher to Mcl-1ES isoform in oral tumors (data not shown). Similarly, western blot analysis showed high Mcl-1L protein expression in oral tumors versus adjacent normal tissues (figure 2b).

### Correlation of Mcl-1 expression with clinicopathological parameters & outcome

Univariate analysis revealed significant correlation of high Mcl-1L expression with node positivity (p = 0.021) and advanced tumors (p = 0.013). However, no significant correlation was observed between expression of Mcl-1 isoforms and gender, age, tobacco/alcohol habits, primary site & differentiation of oral cancer patients (Table 2). Notably, low expression of pro-apoptotic Mcl-1S isoform showed a trend towards positive significance with poorly differentiated tumors (p = 0.053).

**Table 2:** Correlation of Mcl-1 isoform expression & clinicopathological parameters in oral cancer patients.

Parameters	Total	Mcl-1L expression		P-value	Mcl-1S expression		P-value
		Low	High		Low	High	
**Gender**							
Male	99	35	64	*0.446*	69	30	*0.370*
Female	31	12	19		20	11	
**Age** (Years)							
>53	63	27	36	*0.087*	47	16	*0.101*
<53	67	20	47		42	25	
**Habits**							
Tobacco chewing	81	29	52	*0.513*	54	27	*0.323*
Tobacco in any form/In combination	41	15	26		29	12	
No habits	6	3	3		4	2	
NA	2	0	2		2	0	
**Site of tumor**							
Alveolus	35	10	25	*0.925*	20	15	*0.802*
Buccal Mucosa	46	18	28		33	13	
Tongue	45	17	28		33	12	
Other	4	2	2		3	1	
**Tumor size** (T)							
T1 + T2	42	37	5	0.013*	19	14	*0.091*
T3 + T4	88	10	78		70	23	
**Lymph Node** (N)							
Negative	47	38	9	0.021*	31	16	*0.785*
Positive	83	9	74		58	25	
**Differentiation**							
Well	16	7	9	*0.652*	7	9	0.053
Moderate	82	29	53		55	27	
Poor	31	11	20		26	5	

Abbreviations: Mcl-1L  =  Myeloid Cell leukemia-1 **L**ong isoform; Mcl-1S  =  Myeloid cell leukemia-1 **S**hort isoform; NA  =  Not Available; ‘*’ *P*<0.05 value; Bold value indicates a significant difference. High Mcl-1L expression was positively correlated with tumor size, node positivity.

### Correlation of Mcl-1 expression with overall survival

**Figure 3:**
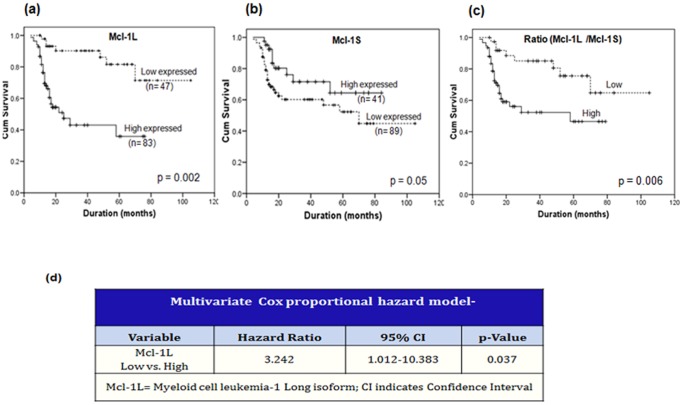
Survival analysis of oral cancer patients. (a-c) Kaplan–Meier estimates of overall survival of oral cancer patients with low or high expression of Mcl-1 isoforms; (d) Multivariate analysis of oral cancer patients.

The Kaplan–Meier survival curves of low and high expressers of Mcl-1L showed a statistically significant difference (p = 0.002). Patients expressing high anti-apoptotic Mcl-1L exhibited poor overall survival versus those expressing low Mcl-1L (Figure 3a). Inversely, patients expressing high pro-apoptotic Mcl-1S exhibited significantly better overall survival (p = 0.051) as compared to those having low Mcl-1S (Figure 3b). Notably, the relative ratio of Mcl-1L/Mcl-1S also showed a positive correlation (p = 0.006) with the poor overall survival of oral cancer patients (Figure 3c). Additionally, the univariate analysis also revealed poor overall survival in node positive versus node negative oral cancer patients (p = 0.003). The other parameters like age, tobacco/alcohol habits and differentiation did not significantly influence overall survival of these patients.

### Multivariate analysis

Among the two isoforms (Mcl-1L & Mcl-1S) analyzed, theMcl-1L expression influenced the overall survival of oral cancer patients. The Mcl-1L variable, which had emerged significant in the univariate analysis, was examined using the Cox regression model in the multivariate analysis (Figure3d). Patients expressing high Mcl-1L exhibited shorter overall survival and 3.2 time's higher risk of poor survival as compared to those expressing low Mcl-1L (p = 0.037). This implies that Mcl-1L is an independent prognostic factor for oral cancer.

### shRNA mediated down regulation of Mcl-1L

**Figure 4:**
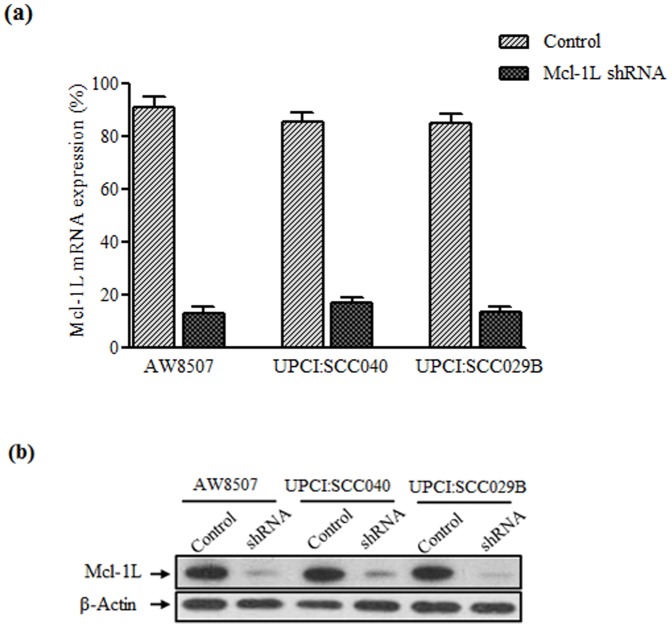
Down regulation of Mcl-1L expression in oral cancer cell lines. (a & b) shRNA mediated down regulation of Mcl-1L mRNA & protein in AW8507, UPCI:SCC040 & SCCC29B oral cancer cells as compared to the control.

In the three oral cancer cell lines (AW8507, UPCI:SCC040 & UPCI:SCC029B) transduced with Mcl-1L shRNA-pTRIPZ lentiviral particles, the Mcl-1L expression was successfully downregulated post doxycycline treatment. The control wells represent the cells without doxycycline treatment. The quantitative real time PCR and western blot analysis confirmed the down regulation of Mcl-1L at both mRNA and protein levels, in all the three cancer cell lines (Figure 4). However, the levels of Mcl-1S & Mcl-1ES remained unaltered (Data not shown).

### Effect of Mcl-1L knockdown &/or Cisplatin on cell death

**Figure 5:**
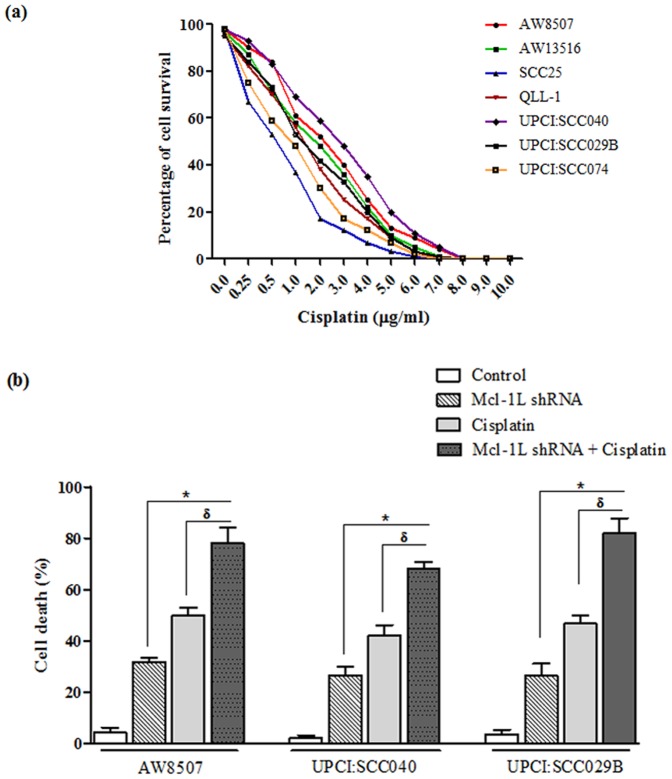
Estimation of Cisplatin IC50 for seven oral cancer cell lines by MTT assay (a) and the effect of Mcl-1L knockdown and/or Cisplatin on induction of cell death by PI staining (b) (* & ^δ^ P<0.05, vs. individual treatments).

The induction of cell death was analyzed by PI staining followed by FACS analysis in the three oral cancer cell lines (AW8507, UPCI:SCC040 & UPCI:SCC029B) post different treatments as described earlier. The shRNA mediated knockdown of abundantly expressed Mcl-1L isoform or Cisplatin treatment revealed induction of cell death in all cell lines. The cell death induced in AW8507, UPCI:SCC040 & UPCI:SCC29B, post Mcl-1L knockdown was 31% (SD 2.5), 27% (SD 1.5) and 24% (SD 3.5) or post Cisplatin treatment was 50% (SD 2.1), 42% (SD 2.7), and 46% (SD 3.1) respectively. Moreover, shRNA mediated Mcl-1L knockdown followed by Cisplatin treatment together showed 78% (SD, 1.2), 71% (SD 1.8) and 82% (SD 2.0) of cell death respectively. The combined treatments of Mcl-1L knockdown and Cisplatin showed a statistically significant (p<0.05) difference in induction of cell death as compared to either treatments alone (Figure 5b), thereby, suggesting a synergistic effect of the combined treatments on cell death.

### Effect of BH3 mimetic Obatoclax and/or Cisplatin on cell viability and growth

**Figure 6:**
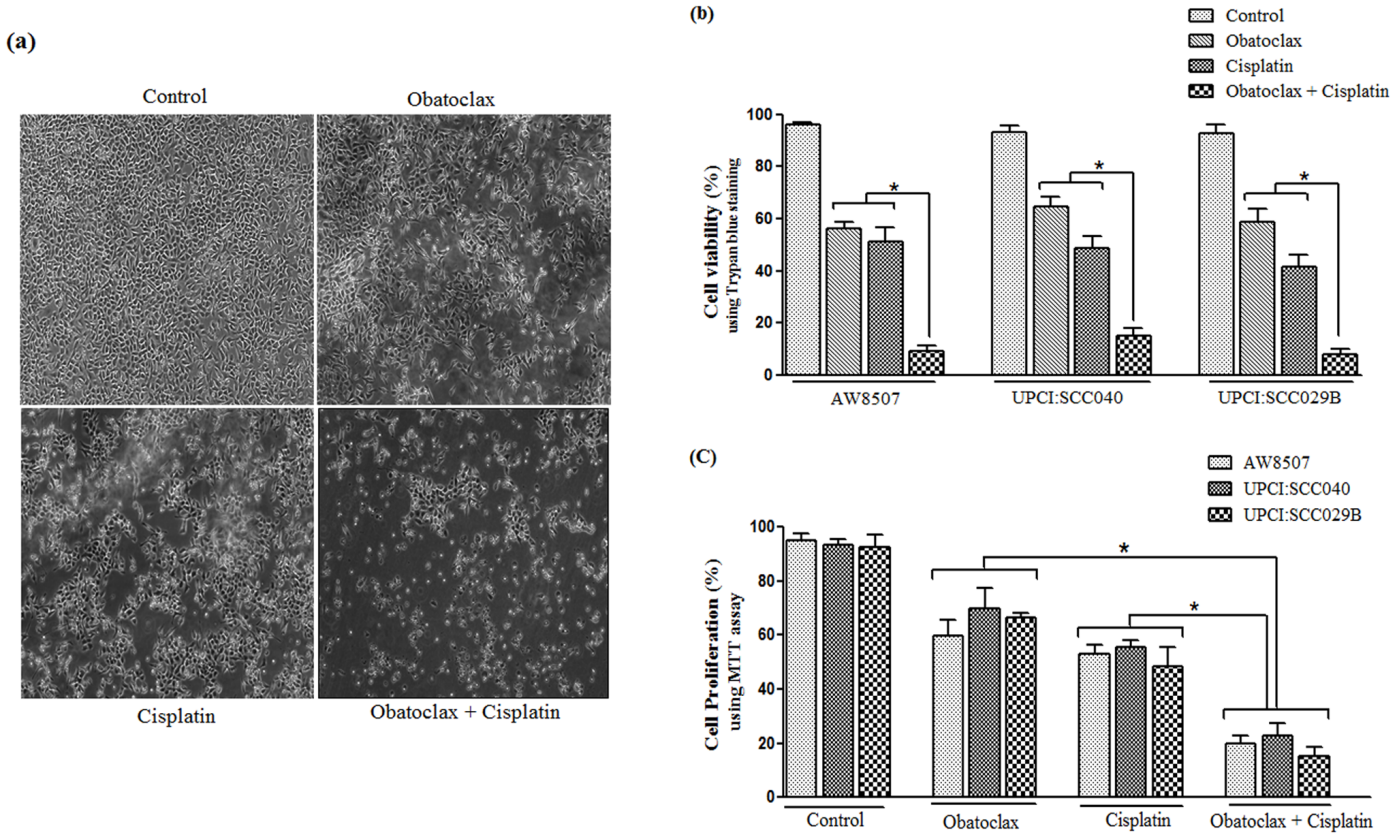
Effect of combination of BH3 mimetic Obatoclax and Cisplatin on cell viability & growth. (a) Confocal microscopic representative images of AW8507 cells post different treatments; (b) Assessment of percent cell viability of oral cancer cells post different treatments by trypan blue dye exclusion assay (* P<0.05, vs. individual treatments); (c) Analysis of proliferation of oral cancer cells after different treatments by MTT assay. (* P<0.05, vs. individual treatments).

Figure 6a illustrates representative confocal microscopy images of AW8507 cells treated with Cisplatin and/or Obatoclax. Interestingly the combined treatments of Obatoclax & Cisplatin showed increased cell death as compared to individual treatments. Additionally, the trypan blue dye exclusion assay was performed to determine the effect on cell viability in oral cancer cell lines (AW8507, UPCI:SCC040 & UPCI:SCC029B) after different treatments. Notably, the BH3 mimetic Obatoclax or Cisplatin could successfully reduce cell viability in all the three cancer cell lines. Moreover, the combination of Obatoclax and Cisplatin together significantly (p<0.05) reduced cell viability as compared to either treatments alone. The combined treatment of Obatoclax & Cisplatin exhibited a 3–7 fold reduction in cell viability as compared to the individual treatments (Figure 6b). Moreover, the MTT proliferation assay also revealed a significant (p<0.05) reduction in cell growth (2–4 fold) after combined treatment of Cisplatin and Obatoclax as compared to either treatments alone (Figure 6c) thereby, suggesting a synergistic effect of the combined treatment on cell viability and growth of oral cancer cells.

## Discussion

In the present study, we assessed the expression of Mcl-1 isoforms (Mcl-1L, Mcl-1S & Mcl-1ES) in oral tumors versus normal tissues, to determine whether they would be useful as prognostic markers in oral cancer patients. So far, limited information is available on the role of Mcl-1 isoforms in pathogenesis of oral cancer. To our knowledge, this is the first study to correlate the expression of the three Mcl-1 isoforms with clinicopathological parameters of oral cancer patients. Our study demonstrates significant high expression of anti-apoptotic Mcl-1L in majority of oral cancer cell lines and tumors (64%) as compared to corresponding normals. We also demonstrate that high Mcl-1L expression was significantly associated with the poor outcome of oral cancer patients. Further, targeting Mcl-1L by shRNA or Obatoclax in combination with Cisplatin could synergistically induce cell death in oral cancer cells. Hence, overexpression of Mcl-1L may represent an important mechanism contributing to the oral cancer cell survival, thereby contributing in the development and progression of oral cancer.

The dysregulation of apoptosis regulating genes has been reported to play a key role in the development and progression of several human malignancies. The anti-apoptotic Mcl-1 is an important member of the apoptosis regulating Bcl-2 family and has been shown to be overexpressed in variety of cancers including, cervical, ovarian, pancreatic, hepatocellular, non-small cell lung, testicular germ cell cancers and melanomas [8]. Notably, the present study for the first time demonstrates overexpression of Mcl-1L splice variant in oral cancer cell lines & majority of oral tumors versus normal tissues. There is only a single report analyzing Mcl-1 isoforms & their clinical significance so far in clear cell renal carcinomas [31]. In contrast to our results, this study however demonstrated an association of low Mcl-1L expression with aggressive phenotypes in clear cell renal carcinomas. Also, there is no information available on the expression of Mcl-1 isoforms in oral cancers. In the present study, the correlation of Mcl-1 isoforms and clinicopathological parameters of oral cancer patients, revealed significant association of Mcl-1L splice variant with advanced tumor size (p = 0.013) and lymph node positivity (p = 0.021). Kaplan–Meier survival analysis of low versus high expressers of Mcl-1L mRNA showed that high Mcl-1L expression was significantly associated with poor overall survival (p = 0.002). As observed in the present study for Mcl-1 isoforms, high Mcl-1 protein expression and its association with poor prognosis has been demonstrated in cervical, gastric, lung and ovarian cancers [11]–[13], [32]. However, a previous study from our lab could not find the above association at the Mcl-1 protein level [14]. The present study was undertaken to elucidate the expression profile of Mcl-1 isoforms in oral cell lines and a large cohort of tumors, which demonstrated the association of anti-apoptotic Mcl-1L expression with prognosis in oral cancer. This is the first study, demonstrating the correlation of Mcl-1L splice variant with outcome of oral cancer patients and Mcl-1L as an independent prognostic marker for oral cancers.

Mcl-1 is tightly regulated at the transcriptional, post transcriptional and translational levels [6] and the exact mechanism of Mcl-1 overexpression in oral cancers is not known. The pro-apoptotic Mcl-1S & Mcl-1ES isoforms were expressed at low or undetectable levels as compared to the predominantly expressed Mcl-1L and did not correlate with clinicopathological parameters of oral cancer patients. Notably, the short pro-apoptotic Mcl-1S only binds to Mcl-1L possibly neutralizing its anti-apoptotic function [33]. Moreover, the recently identified pro-apoptotic Mcl-1ES isoform may also bind and neutralize the anti-apoptotic function of full length Mcl-1L isoform, though it is expressed at very low or undetectable levels. We therefore analyzed the relative ratios of Mcl-1L/Mcl-1S isoforms, which revealed a significant positive correlation with the poor overall survival of patients, implying that the low expression of Mcl-1S & Mcl-1ES in oral cancers is possibly insufficient to completely neutralize the high expression of anti-apoptotic Mcl-1L isoform. To the best of our knowledge this is the first study quantitating the expression of the three Mcl-1 isoforms and their correlation with clinicopathological parameters & outcome of oral cancer patients. Our findings are also supported by the previous reports in gastric and cervical cancers demonstrating the association between high Mcl-1 protein expression with tumor size, histological grade, lymph node involvement, metastasis & poor clinical outcome [11], [34]. Moreover, the prognostic significance of high Mcl-1 protein expression has also been demonstrated in breast, ovarian, non-small cell lung cancer and several hematological malignancies [10], [13], [35].

Mcl-1 is overexpressed in a variety of human malignancies and proposed to be a potential therapeutic target [8]. Mcl-1 overexpression also appears to be a key factor in the resistance of various cancer types to conventional treatments, including radiation and chemotherapy. The down regulation of Mcl-1 has been shown to sensitize neuroblastoma cells to cytotoxic chemotherapy and resistant melanoma cells to Fas mediated apoptosis [36], [37]. Moreover, in our present study, shRNA mediated down regulation of Mcl-1L has shown to chemosensitize oral cancer cells (AW8507, UPCI:SCC040 & UPCI:SCC029B) to Cisplatin indicating a crucial role for Mcl-1 in treatment resistance in oral cancers. Studies targeting Mcl-1 via antisense therapy have also shown to chemosensitize hepatocellular carcinoma cells *in vitro* [38], [39]. Although our study indicates depletion of Mcl-1L expression from 90% to 20% by shRNA (fig.4a) a minimal cell death (25%) was observed. This suggests that besides Mcl-1 there might be other factors contributing in survival of cells post Cisplatin treatment. Several other studies have also reported proteins like Survivin [40], Oct4 & Nanog [41], MUC4 [42], secretary Kin17 [43], etc. playing an important role in chemoresistance of oral squamous carcinoma cells. However, this is the first study correlating the association of Mcl-1L splice variant with chemoresistance of oral cancer.

Recent studies from our lab have shown a role for Mcl-1L isoform in radioresistance of oral squamous carcinoma cells and as a prognostic factor in prediction of disease-free survival of oral cancer patients treated with definitive radiotherapy. [15], [17]. Hence, depletion of Mcl-1 appears to be a prerequisite to radio/chemo sensitize cancer cells that overexpress Mcl-1 protein, to promote apoptosis. Several attempts have been made to target the anti-apoptotic Bcl-2 family proteins including Mcl-1 in tumors using a variety of inhibitors. Among these, the most promising inhibitor, a BH3 mimetic ABT-737 has failed to overcome resistance due to Mcl-1 overexpression. Further studies indicate that, Mcl-1 down-regulation alone can potentiate ABT-737 lethality in leukemia cells [44]. In the present study a pan Bcl-2 small molecule inhibitor, Obatoclax (GX15-070) which is known to antagonize Mcl-1 and overcome Mcl-1 mediated resistance to apoptosis was used [21]. Obatoclax has been shown to sequester anti-apoptotic Bcl-2 family proteins and up-regulate expression of Puma, Noxa & Bim which further induced apoptosis in neoplastic mast cells [45]. In a recent study, Obatoclax has been shown to reduce levels of anti-apoptotic proteins including Mcl-1 and induced pro-survival autophagy & apoptosis in Head & Neck Squamous Carcinoma Cells [46]. This is the only study available supporting the importance of BH3 mimetic small molecule inhibitors in treatment of oral cancer patients. The present study has demonstrated a synergistic effect of Obatoclax and Cisplatin on oral cancer cell viability and growth, indicating that elimination of anti-apoptotic Mcl-1L is crucial for chemosensitization the oral cancer cells.

This is the first study that demonstrates expression of the three Mcl-1 splice variants in oral cancer cell lines & tumor tissues. Moreover, high Mcl-1L mRNA was significantly associated with advanced tumor size, nodal involvement & poor outcome of oral cancer patients. Also, depleting or inhibiting Mcl-1L levels could successfully chemosensitize oral cancer cells to Cisplatin treatment. These studies suggest that Mcl-1L splice variant is overexpressed in oral cancers and its targeting may be important in the treatment outcome of oral cancer patients.

## Conclusion

Our study for the first time assessed Mcl-1 splice variants in oral cancer and demonstrated, anti-apoptotic Mcl-1L isoform to be a chemoresistance and prognostic factor and a potential therapeutic target in oral cancers.

## References

[1] Dikshit R, Gupta PC, Ramasundarahettige C, Gajalakshmi V, Aleksandrowicz L, et al (2012) Cancer mortality in India: a nationally representative survey. Lancet 379: 1807–1816. 22460346 10.1016/S0140-6736(12)60358-4

[2] Coelho KR (2012) Challenges of the oral cancer burden in India. Journal of cancer epidemiology 2012. 10.1155/2012/701932 PMC3471448 23093961

[3] Reed JC (1999) Dysregulation of apoptosis in cancer. Journal of Clinical Oncology 17: 2941–2941. 10561374 10.1200/JCO.1999.17.9.2941

[4] Cory S, Adams JM (2002) The Bcl2 family: regulators of the cellular life-or-death switch. Nature Reviews Cancer 2: 647–656. 12209154 10.1038/nrc883

[5] Craig R (2002) MCL1 provides a window on the role of the BCL2 family in cell proliferation, differentiation and tumorigenesis. Leukemia: official journal of the Leukemia Society of America, Leukemia Research Fund, UK 16: 444. 10.1038/sj.leu.2402416 11960321

[6] Thomas LW, Lam C, Edwards SW (2010) Mcl-1; the molecular regulation of protein function. FEBS letters 584: 2981–2989. 20540941 10.1016/j.febslet.2010.05.061

[7] Willis SN, Chen L, Dewson G, Wei A, Naik E, et al (2005) Proapoptotic Bak is sequestered by Mcl-1 and Bcl-xL, but not Bcl-2, until displaced by BH3-only proteins. Genes & development 19: 1294–1305. 15901672 10.1101/gad.1304105 PMC1142553

[8] Akgul C (2009) Mcl-1 is a potential therapeutic target in multiple types of cancer. Cellular and molecular life sciences 66: 1326–1336. 19099185 10.1007/s00018-008-8637-6 PMC11131550

[9] Nagata M, Wada K, Nakajima A, Nakajima N, Kusayama M, et al (2009) Role of myeloid cell leukemia-1 in cell growth of squamous cell carcinoma. Journal of pharmacological sciences 110: 344–353. 19571464 10.1254/jphs.08339fp

[10] O'Driscoll, Cronin D, Kennedy S, Purcell R, Linehan R, et al (2004) Expression and prognostic relevance of Mcl-1 in breast cancer. Anticancer research 24: 473–482. 15152946

[11] Likui W, Qun L, Wanqing Z, Haifeng S, Fangqiu L, et al (2009) Prognostic role of myeloid cell leukemia-1 protein (Mcl-1) expression in human gastric cancer. Journal of surgical oncology 100: 396–400. 19582795 10.1002/jso.21344

[12] Zhang T, Zhao C, Luo L, Zhao H, Cheng J, et al (2012) The expression of Mcl-1 in human cervical cancer and its clinical significance. Medical Oncology 29: 1985–1991. 21674276 10.1007/s12032-011-0005-y

[13] Shigemasa K, Katoh O, Shiroyama Y, Mihara S, Mukai K, et al (2002) Increased MCL–1 Expression Is Associated with Poor Prognosis in Ovarian Carcinomas. Cancer Science 93: 542–550. 10.1111/j.1349-7006.2002.tb01289.x PMC5927039 12036450

[14] Mallick S, Patil R, Gyanchandani R, Pawar S, Palve V, et al (2009) Human oral cancers have altered expression of Bcl-2 family members and increased expression of the anti-apoptotic splice variant of Mcl-1. The Journal of pathology 217: 398–407. 19009587 10.1002/path.2459

[15] Mallick S, Agarwal J, Kannan S, Pawar S, Kane S, et al (2010) PCNA and anti-apoptotic Mcl-1 proteins predict disease-free survival in oral cancer patients treated with definitive radiotherapy. Oral oncology 46: 688–693. 20729132 10.1016/j.oraloncology.2010.04.003

[16] Kim J-H, Sim S-H, Ha H-J, Ko J-J, Lee K, et al (2009) MCL-1ES, a novel variant of MCL-1, associates with MCL-1L and induces mitochondrial cell death. FEBS letters 583: 2758–2764. 19683529 10.1016/j.febslet.2009.08.006

[17] Palve VC, Teni TR (2012) Association of anti-apoptotic Mcl-1L isoform expression with radioresistance of oral squamous carcinoma cells. Radiation Oncology 7: 1–11. 22873792 10.1186/1748-717X-7-135 PMC3487741

[18] Yang C, Kaushal V, Shah SV, Kaushal GP (2007) Mcl-1 is downregulated in cisplatin-induced apoptosis, and proteasome inhibitors restore Mcl-1 and promote survival in renal tubular epithelial cells. American Journal of Physiology-Renal Physiology 292: F1710–F1717. 17311906 10.1152/ajprenal.00505.2006

[19] Simonin K, Brotin E, Dufort S, Dutoit S, Goux D, et al (2009) Mcl-1 is an important determinant of the apoptotic response to the BH3-mimetic molecule HA14-1 in cisplatin-resistant ovarian carcinoma cells. Molecular cancer therapeutics 8: 3162–3170. 19887550 10.1158/1535-7163.MCT-09-0493

[20] Zhang H, Guttikonda S, Roberts L, Uziel T, Semizarov D, et al (2010) Mcl-1 is critical for survival in a subgroup of non-small-cell lung cancer cell lines. Oncogene 30: 1963–1968. 21132008 10.1038/onc.2010.559

[21] Nguyen M, Marcellus RC, Roulston A, Watson M, Serfass L, et al (2007) Small molecule obatoclax (GX15-070) antagonizes MCL-1 and overcomes MCL-1-mediated resistance to apoptosis. Proceedings of the National Academy of Sciences 104: 19512–19517. 10.1073/pnas.0709443104 PMC2148320 18040043

[22] Quinn BA, Dash R, Azab B, Sarkar S, Das SK, et al (2011) Targeting Mcl-1 for the therapy of cancer. Expert opinion on investigational drugs 20: 1397–1411. 21851287 10.1517/13543784.2011.609167 PMC3205956

[23] Park HR, Kim SH, Lee SY, Sung JM, Park AR, et al (2011) Nuclear localization of Nm23-H1 in head and neck squamous cell carcinoma is associated with radiation resistance. Cancer 117: 1864–1873. 21509763 10.1002/cncr.25760

[24] Rheinwald JG, Beckett MA (1981) Tumorigenic keratinocyte lines requiring anchorage and fibroblast support cultured from human squamous cell carcinomas. Cancer research 41: 1657–1663. 7214336

[25] Martin CL, Reshmi SC, Ried T, Gottberg W, Wilson JW, et al (2008) Chromosomal imbalances in oral squamous cell carcinoma: examination of 31 cell lines and review of the literature. Oral oncology 44: 369–382. 17681875 10.1016/j.oraloncology.2007.05.003 PMC2362065

[26] Tatake RJ, Rajaram N, Damle R, Balsara B, Bhisey A, et al (1990) Establishment and characterization of four new squamous cell carcinoma cell lines derived from oral tumors. Journal of cancer research and clinical oncology 116: 179–186. 1691185 10.1007/BF01612674 PMC12200902

[27] Raul U, Sawant S, Dange P, Kalraiya R, Ingle A, et al (2004) Implications of cytokeratin 8/18 filament formation in stratified epithelial cells: induction of transformed phenotype. Int J Cancer 111: 662–668. 15252834 10.1002/ijc.20349

[28] Chang SE, Foster S, Betts D, Marnock WE (1992) DOK, a cell line established from human dysplastic oral mucosa, shows a partially transformed non-malignant phenotype. International journal of cancer 52: 896–902. 1459732 10.1002/ijc.2910520612

[29] Martin CL, Reshmi SC, Ried T, Gottberg W, Wilson JW, et al (2008) Chromosomal imbalances in oral squamous cell carcinoma. Examination of 31 cell lines and review of the literature. Oral oncology 44: 369. 17681875 10.1016/j.oraloncology.2007.05.003 PMC2362065

[30] Schmittgen TD, Livak KJ (2008) Analyzing real-time PCR data by the comparative CT method. Nature protocols 3: 1101–1108. 18546601 10.1038/nprot.2008.73

[31] Kempkensteffen C, Hinz S, Johannsen M, Krause H, Magheli A, et al (2009) Expression of Mcl-1 splicing variants in clear-cell renal cancer and their correlation with histopathological parameters and prognosis. Tumor Biology 30: 73–79. 19401626 10.1159/000215826

[32] Luo L, Zhang T, Liu H, Lv T, Yuan D, et al (2012) MiR-101 and Mcl-1 in non-small-cell lung cancer: expression profile and clinical significance. Medical Oncology 29: 1681–1686. 21993632 10.1007/s12032-011-0085-8

[33] Bae J, Leo CP, Hsu SY, Hsueh AJ (2000) MCL-1S, a splicing variant of the antiapoptotic BCL-2 family member MCL-1, encodes a proapoptotic protein possessing only the BH3 domain. J Biol Chem 275: 25255–25261. 10837489 10.1074/jbc.M909826199

[34] Zhang T, Zhao C, Luo L, Zhao H, Cheng J, et al.. (2012) The expression of Mcl-1 in human cervical cancer and its clinical significance. Medical Oncology: 1–7. 10.1007/s12032-011-0005-y 21674276

[35] Pepper C, Lin TT, Pratt G, Hewamana S, Brennan P, et al (2008) Mcl-1 expression has in vitro and in vivo significance in chronic lymphocytic leukemia and is associated with other poor prognostic markers. Blood 112: 3807–3817. 18599795 10.1182/blood-2008-05-157131

[36] Lestini BJ, Goldsmith KC, Fluchel MN, Liu X, Chen NL, et al (2009) Mcl1 downregulation sensitizes neuroblastoma to cytotoxic chemotherapy and small molecule Bcl2-family antagonists. Cancer biology & therapy 8: 1587–1595. 19556859 10.4161/cbt.8.16.8964 PMC3770183

[37] Chetoui N, Sylla K, Gagnon-Houde J-V, Alcaide-Loridan C, Charron D, et al (2008) Down-regulation of mcl-1 by small interfering RNA sensitizes resistant melanoma cells to fas-mediated apoptosis. Molecular Cancer Research 6: 42–52. 18234961 10.1158/1541-7786.MCR-07-0080

[38] Schulze-Bergkamen H, Fleischer B, Schuchmann M, Weber A, Weinmann A, et al (2006) Suppression of Mcl-1 via RNA interference sensitizes human hepatocellular carcinoma cells towards apoptosis induction. BMC cancer 6: 232. 17014711 10.1186/1471-2407-6-232 PMC1601962

[39] Sieghart W, Losert D, Strommer S, Cejka D, Schmid K, et al (2006) Mcl-1 overexpression in hepatocellular carcinoma: a potential target for antisense therapy. Journal of hepatology 44: 151–157. 16289418 10.1016/j.jhep.2005.09.010

[40] Liping S, Ying W, Mingzhen X, Yuan L, Lei Y (2010) Up-regulation of survivin in oral squamous cell carcinoma correlates with poor prognosis and chemoresistance. Oral Surgery, Oral Medicine, Oral Pathology, Oral Radiology, and Endodontology 110: 484–491. 10.1016/j.tripleo.2010.04.009 20868995

[41] Tsai LL, Yu CC, Chang YC, Yu CH, Chou MY (2011) Markedly increased Oct4 and Nanog expression correlates with cisplatin resistance in oral squamous cell carcinoma. Journal of Oral Pathology & Medicine 40: 621–628. 21342274 10.1111/j.1600-0714.2011.01015.x

[42] Hamada T (2014) Expression of MUC4 is correlated with chemoresistance and survival in oral squamous cell carcinoma. 2014 Annual Meeting. Aaoms.

[43] Liu X, Deng L, Zhang H, Zeng T, Wang H, et al (2014) Secretory Kin17 is Correlated with Chemoresistance in Oral Squamous Cell Carcinoma. Journal of Analytical Oncology 3: 18–25.

[44] Chen S, Dai Y, Harada H, Dent P, Grant S (2007) Mcl-1 down-regulation potentiates ABT-737 lethality by cooperatively inducing Bak activation and Bax translocation. Cancer research 67: 782–791. 17234790 10.1158/0008-5472.CAN-06-3964

[45] Peter B, Cerny-Reiterer S, Hadzijusufovic E, Schuch K, Stefanzl G, et al.. (2013) The pan-Bcl-2 blocker obatoclax promotes the expression of Puma, Noxa, and Bim mRNA and induces apoptosis in neoplastic mast cells. Journal of leukocyte biology. 10.1189/jlb.1112609 24052572

[46] Yazbeck VY, Li C, Grandis JR, Zang Y, Johnson DE (2014) Single-agent obatoclax (GX15-070) potently induces apoptosis and pro-survival autophagy in head and neck squamous cell carcinoma cells. Oral oncology 50: 120–127. 24216166 10.1016/j.oraloncology.2013.10.013 PMC3944051

